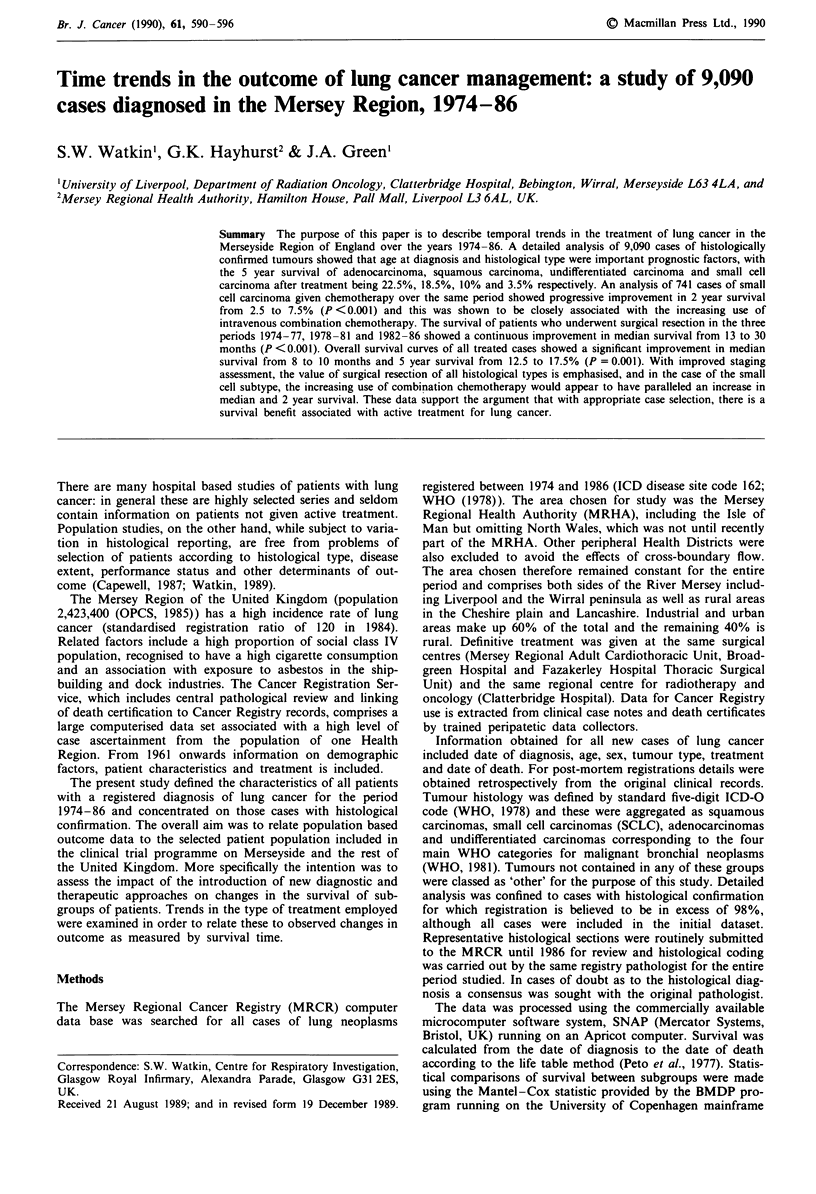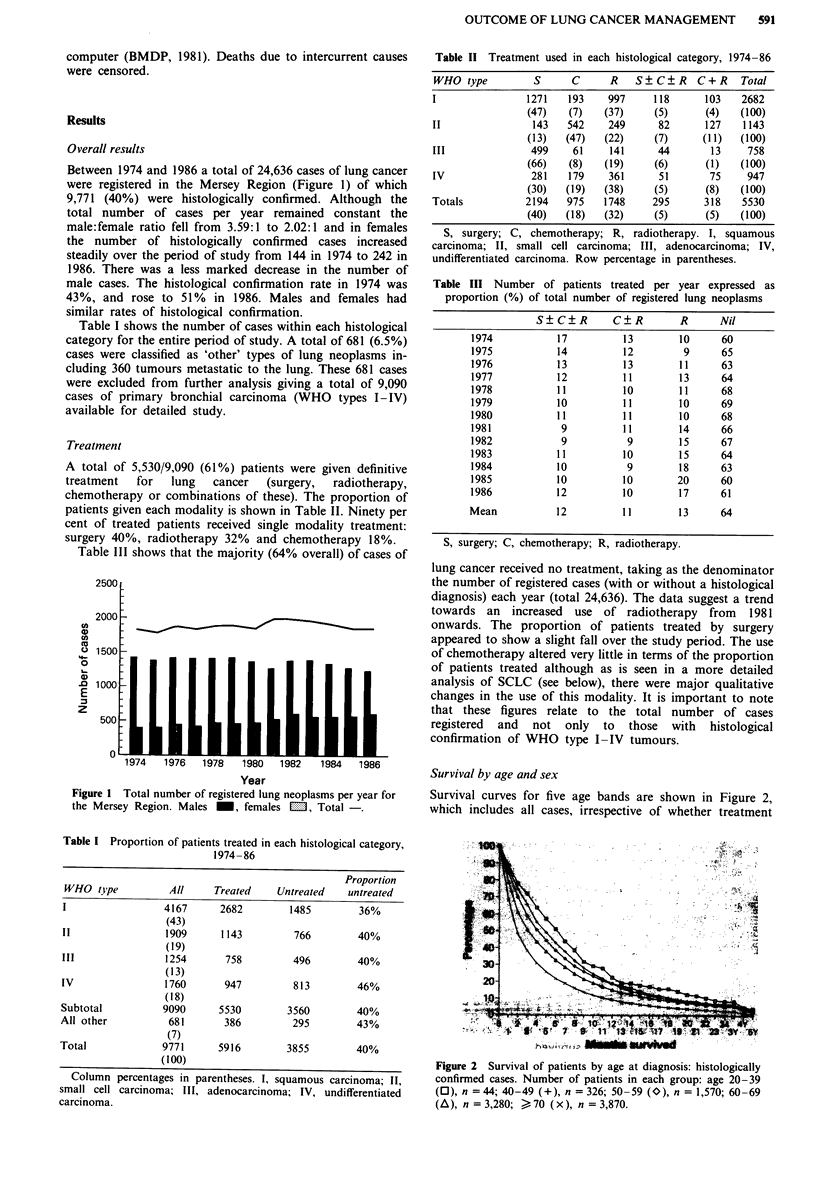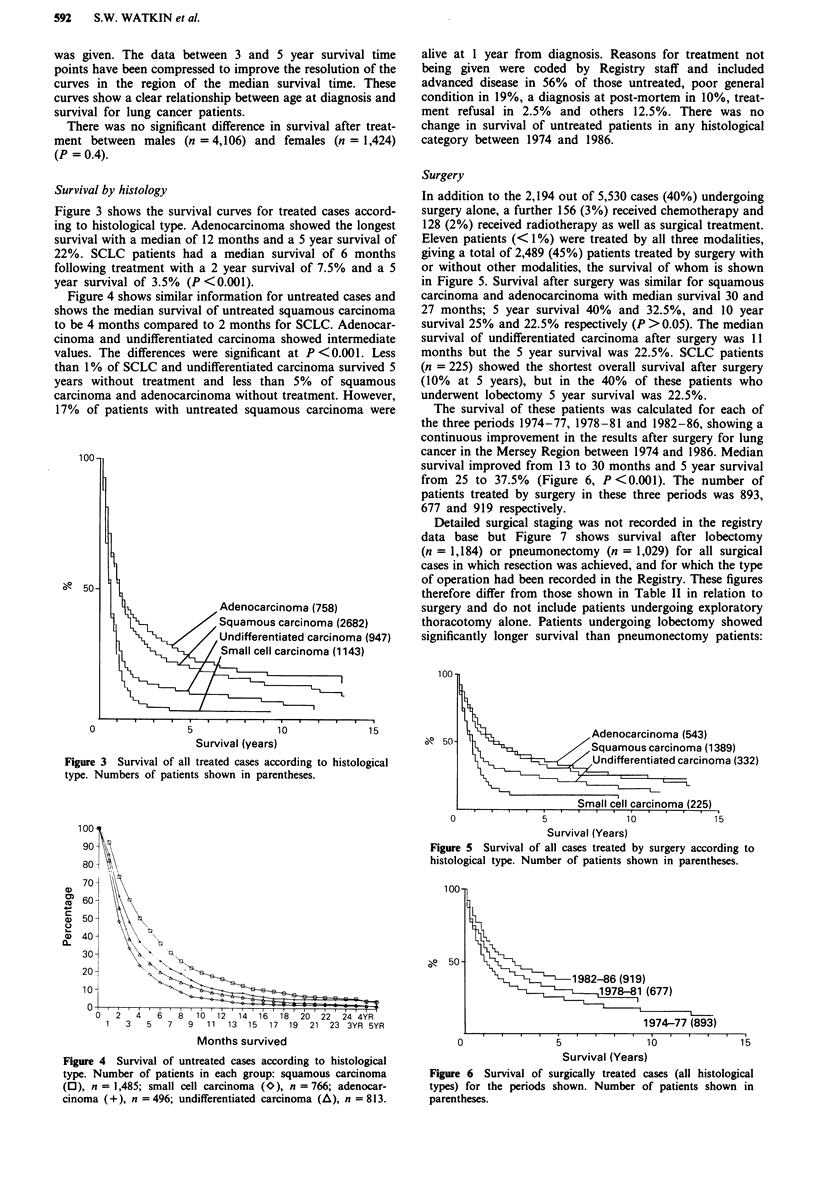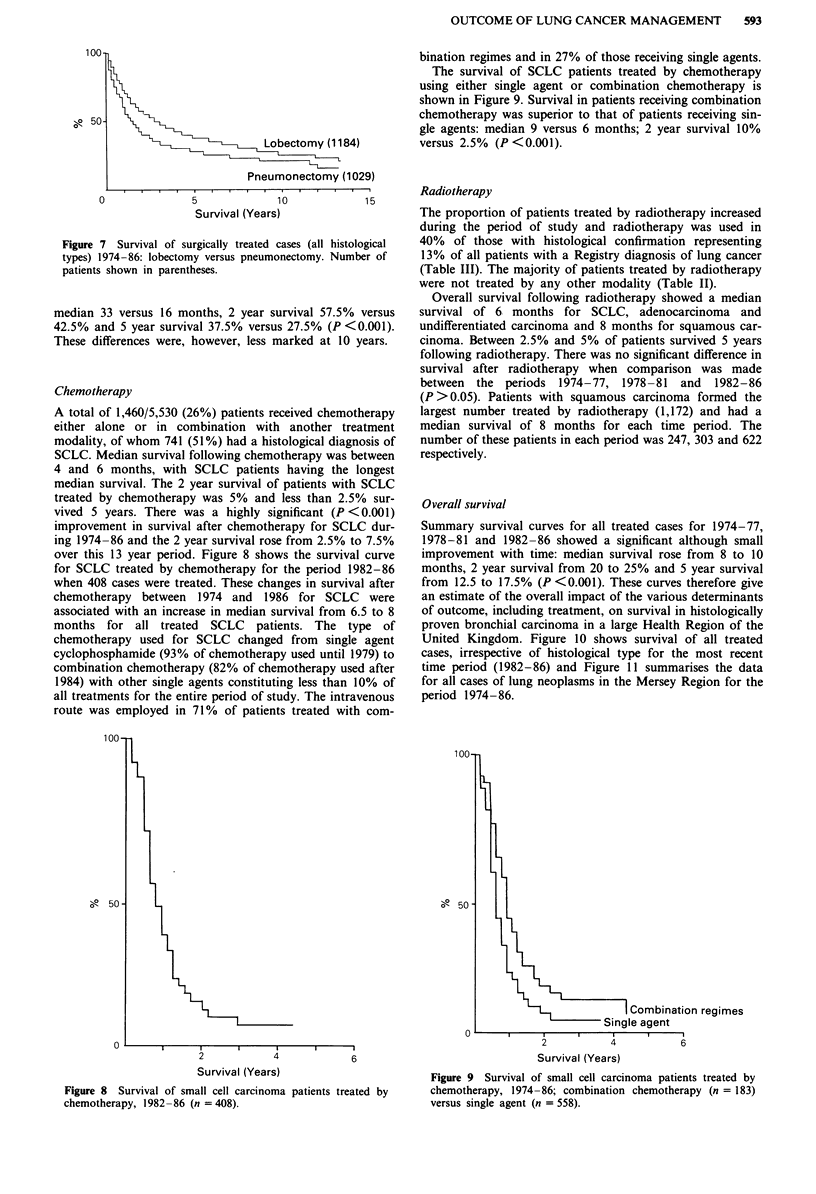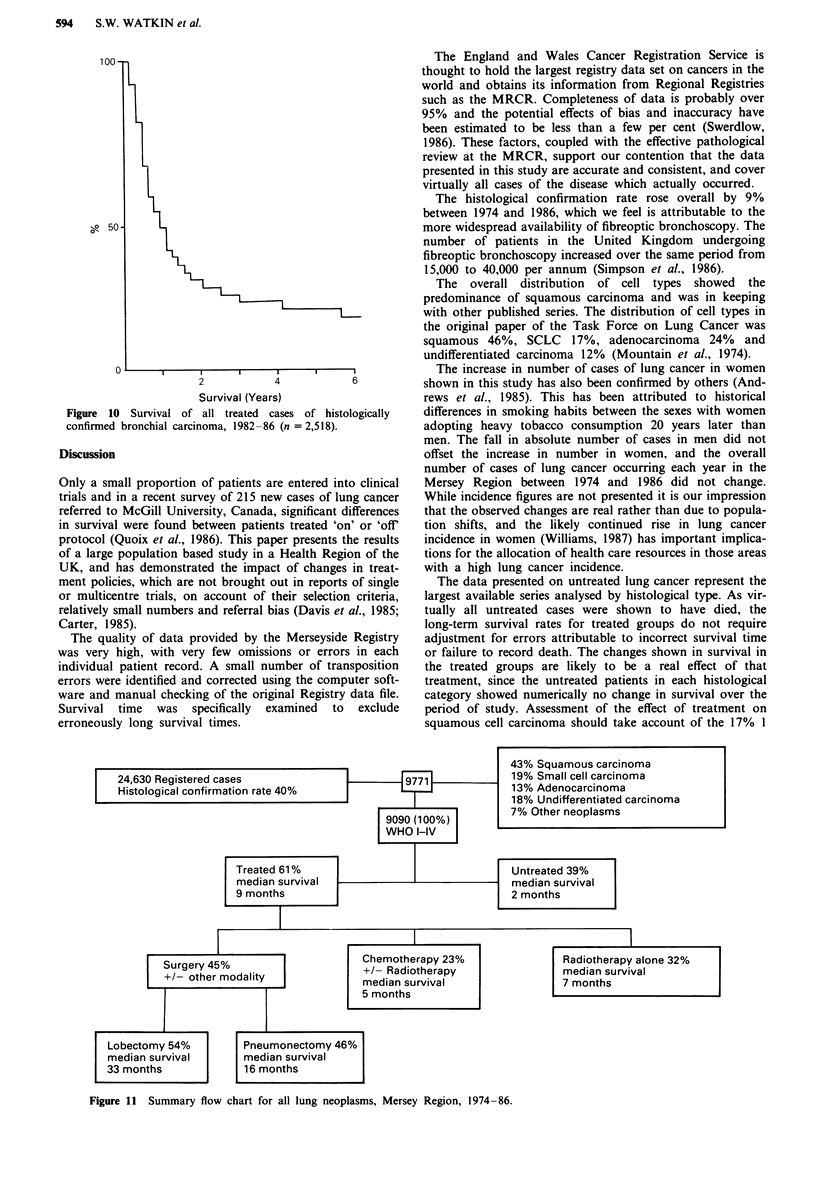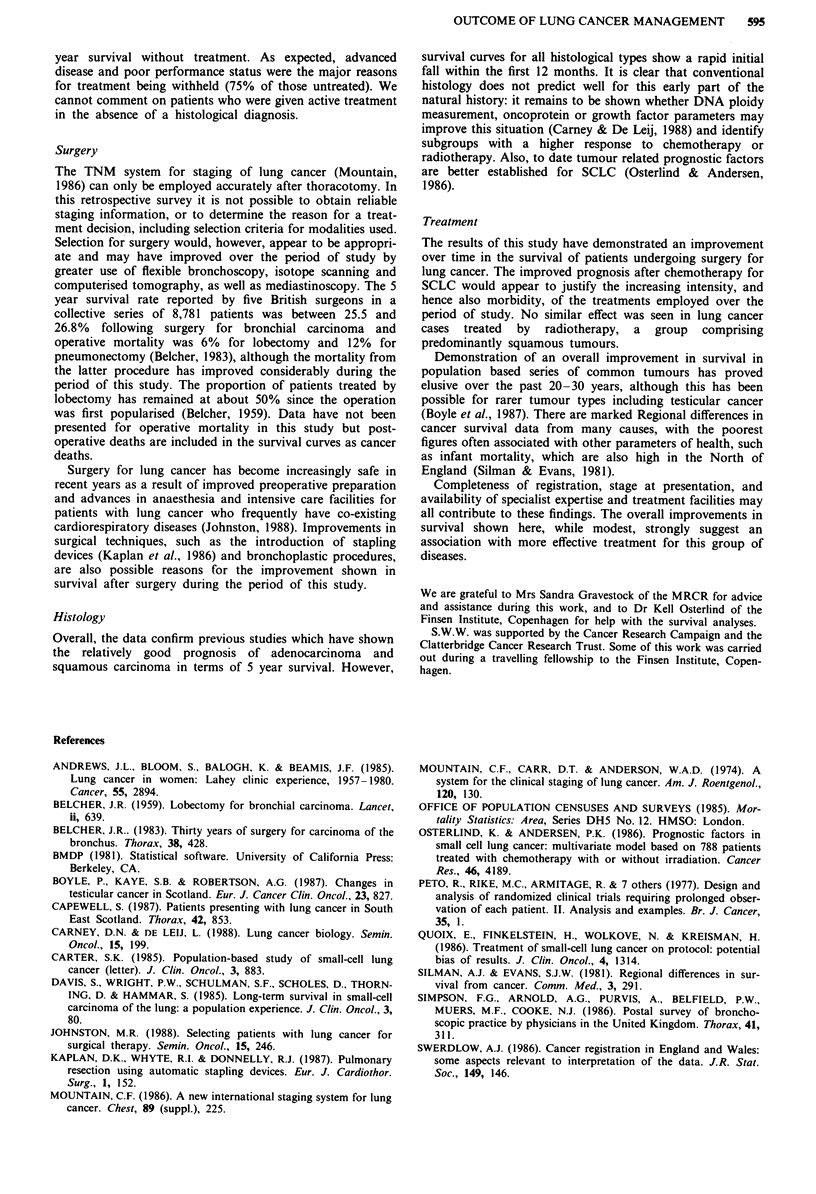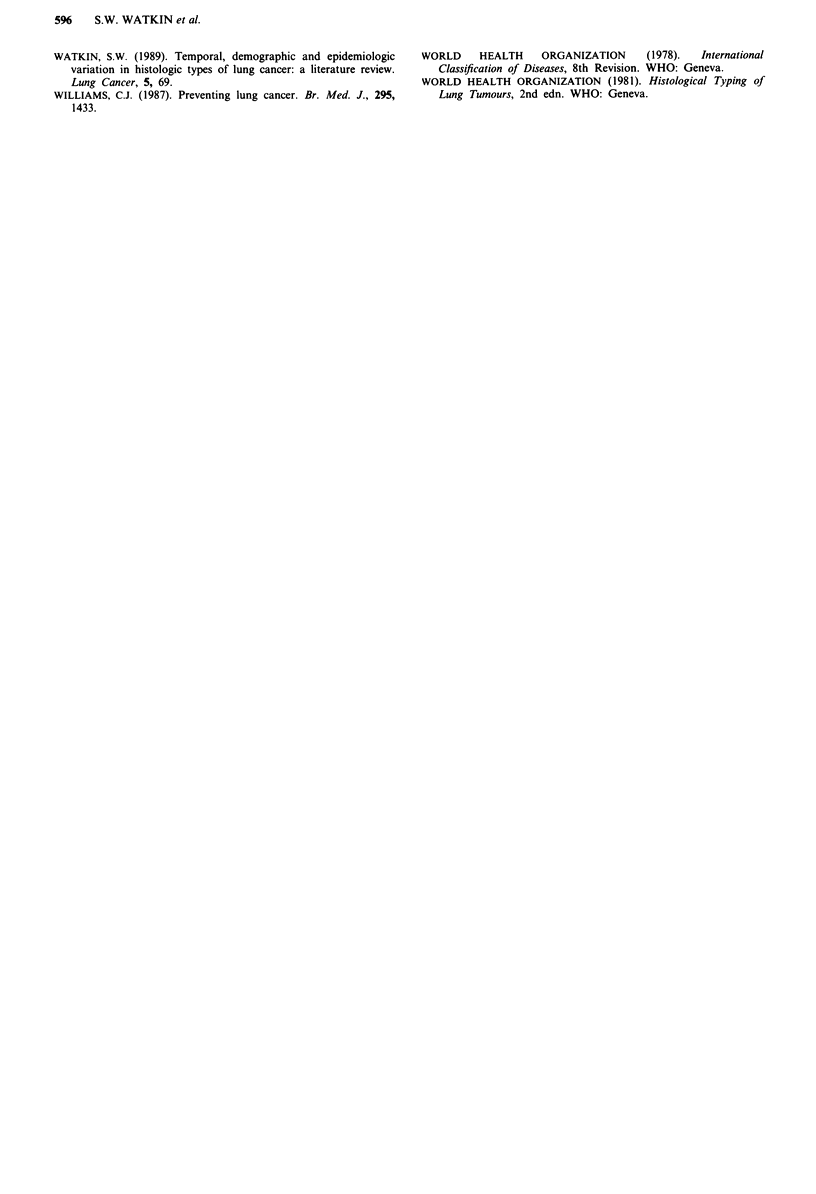# Time trends in the outcome of lung cancer management: a study of 9,090 cases diagnosed in the Mersey Region, 1974-86.

**DOI:** 10.1038/bjc.1990.132

**Published:** 1990-04

**Authors:** S. W. Watkin, G. K. Hayhurst, J. A. Green

**Affiliations:** University of Liverpool, Department of Radiation Oncology, Clatterbridge Hospital, Wirral, Merseyside, UK.

## Abstract

The purpose of this paper is to describe temporal trends in the treatment of lung cancer in the Merseyside Region of England over the years 1974-86. A detailed analysis of 9,090 cases of histologically confirmed tumours showed that age at diagnosis and histological type were important prognostic factors, with the 5 year survival of adenocarcinoma, squamous carcinoma, undifferentiated carcinoma and small cell carcinoma after treatment being 22.5%, 18.5%, 10% and 3.5% respectively. An analysis of 741 cases of small cell carcinoma given chemotherapy over the same period showed progressive improvement in 2 year survival from 2.5 to 7.5% (P less than 0.001) and this was shown to be closely associated with the increasing use of intravenous combination chemotherapy. The survival of patients who underwent surgical resection in the three periods 1974-77, 1978-81 and 1982-86 showed a continuous improvement in median survival from 13 to 30 months (P less than 0.001). Overall survival curves of all treated cases showed a significant improvement in median survival from 8 to 10 months and 5 year survival from 12.5 to 17.5% (P = 0.001). With improved staging assessment, the value of surgical resection of all histological types is emphasised, and in the case of the small cell subtype, the increasing use of combination chemotherapy would appear to have paralleled an increase in median and 2 year survival. These data support the argument that with appropriate case selection, there is a survival benefit associated with active treatment for lung cancer.


Br. J. Cancer (1990), 61, 590-596 © Macmillan Press Ltd., 1990

### Time trends in the outcome of lung cancer management: a study of 9,090 cases diagnosed in the Mersey Region, 1974-86

S.W. Watkin[1], G.K. Hayhurst[2] & J.A. Green[1]

[1]*University of Liverpool, Department of Radiation Oncology, Clatterbridge Hospital, Bebington, Wirral, Merseyside L63 4LA, and* [2]*Mersey Regional Health Authority, Hamilton House, Pall Mall, Liverpool L3 6AL, UK.*

**Summary** The purpose of this paper is to describe temporal trends in the treatment of lung cancer in the Merseyside Region of England over the years 1974-86. A detailed analysis of 9,090 cases of histologically confirmed tumours showed that age at diagnosis and histological type were important prognostic factors, with the 5 year survival of adenocarcinoma, squamous carcinoma, undifferentiated carcinoma and small cell carcinoma after treatment being 22.5%, 18.5%, 10% and 3.5% respectively. An analysis of 741 cases of small cell carcinoma given chemotherapy over the same period showed progressive improvement in 2 year survival from 2.5 to 7.5% ($P < 0.001$) and this was shown to be closely associated with the increasing use of intravenous combination chemotherapy. The survival of patients who underwent surgical resection in the three periods 1974-77, 1978-81 and 1982-86 showed a continuous improvement in median survival from 13 to 30 months ($P < 0.001$). Overall survival curves of all treated cases showed a significant improvement in median survival from 8 to 10 months and 5 year survival from 12.5 to 17.5% ($P = 0.001$). With improved staging assessment, the value of surgical resection of all histological types is emphasised, and in the case of the small cell subtype, the increasing use of combination chemotherapy would appear to have paralleled an increase in median and 2 year survival. These data support the argument that with appropriate case selection, there is a survival benefit associated with active treatment for lung cancer.

There are many hospital based studies of patients with lung cancer: in general these are highly selected series and seldom contain information on patients not given active treatment. Population studies, on the other hand, while subject to variation in histological reporting, are free from problems of selection of patients according to histological type, disease extent, performance status and other determinants of outcome (Capewell, 1987; Watkin, 1989).

The Mersey Region of the United Kingdom (population 2,423,400 (OPCS, 1985)) has a high incidence rate of lung cancer (standardised registration ratio of 120 in 1984). Related factors include a high proportion of social class IV population, recognised to have a high cigarette consumption and an association with exposure to asbestos in the shipbuilding and dock industries. The Cancer Registration Service, which includes central pathological review and linking of death certification to Cancer Registry records, comprises a large computerised data set associated with a high level of case ascertainment from the population of one Health Region. From 1961 onwards information on demographic factors, patient characteristics and treatment is included.

The present study defined the characteristics of all patients with a registered diagnosis of lung cancer for the period 1974-86 and concentrated on those cases with histological confirmation. The overall aim was to relate population based outcome data to the selected patient population included in the clinical trial programme on Merseyside and the rest of the United Kingdom. More specifically the intention was to assess the impact of the introduction of new diagnostic and therapeutic approaches on changes in the survival of subgroups of patients. Trends in the type of treatment employed were examined in order to relate these to observed changes in outcome as measured by survival time.

### Methods

The Mersey Regional Cancer Registry (MRCR) computer data base was searched for all cases of lung neoplasms registered between 1974 and 1986 (ICD disease site code 162; WHO (1978)). The area chosen for study was the Mersey Regional Health Authority (MRHA), including the Isle of Man but omitting North Wales, which was not until recently part of the MRHA. Other peripheral Health Districts were also excluded to avoid the effects of cross-boundary flow. The area chosen therefore remained constant for the entire period and comprises both sides of the River Mersey including Liverpool and the Wirral peninsula as well as rural areas in the Cheshire plain and Lancashire. Industrial and urban areas make up 60% of the total and the remaining 40% is rural. Definitive treatment was given at the same surgical centres (Mersey Regional Adult Cardiothoracic Unit, Broadgreen Hospital and Fazakerley Hospital Thoracic Surgical Unit) and the same regional centre for radiotherapy and oncology (Clatterbridge Hospital). Data for Cancer Registry use is extracted from clinical case notes and death certificates by trained peripatetic data collectors.

Information obtained for all new cases of lung cancer included date of diagnosis, age, sex, tumour type, treatment and date of death. For post-mortem registrations details were obtained retrospectively from the original clinical records. Tumour histology was defined by standard five-digit ICD-O code (WHO, 1978) and these were aggregated as squamous carcinomas, small cell carcinomas (SCLC), adenocarcinomas and undifferentiated carcinomas corresponding to the four main WHO categories for malignant bronchial neoplasms (WHO, 1981). Tumours not contained in any of these groups were classed as 'other' for the purpose of this study. Detailed analysis was confined to cases with histological confirmation for which registration is believed to be in excess of 98%, although all cases were included in the initial dataset. Representative histological sections were routinely submitted to the MRCR until 1986 for review and histological coding was carried out by the same registry pathologist for the entire period studied. In cases of doubt as to the histological diagnosis a consensus was sought with the original pathologist.

The data was processed using the commercially available microcomputer software system, SNAP (Mercator Systems, Bristol, UK) running on an Apricot computer. Survival was calculated from the date of diagnosis to the date of death according to the life table method (Peto *et al.*, 1977). Statistical comparisons of survival between subgroups were made using the Mantel-Cox statistic provided by the BMDP program running on the University of Copenhagen mainframe

Correspondence: S.W. Watkin, Centre for Respiratory Investigation, Glasgow Royal Infirmary, Alexandra Parade, Glasgow G31 2ES, UK.
Received 21 August 1989; and in revised form 19 December 1989.

computer (BMDP, 1981). Deaths due to intercurrent causes were censored.

### Results

### *Overall results*

Between 1974 and 1986 a total of 24,636 cases of lung cancer were registered in the Mersey Region (Figure 1) of which 9,771 (40%) were histologically confirmed. Although the total number of cases per year remained constant the male:female ratio fell from 3.59:1 to 2.02:1 and in females the number of histologically confirmed cases increased steadily over the period of study from 144 in 1974 to 242 in 1986. There was a less marked decrease in the number of male cases. The histological confirmation rate in 1974 was 43%, and rose to 51% in 1986. Males and females had similar rates of histological confirmation.

Table I shows the number of cases within each histological category for the entire period of study. A total of 681 (6.5%) cases were classified as 'other' types of lung neoplasms including 360 tumours metastatic to the lung. These 681 cases were excluded from further analysis giving a total of 9,090 cases of primary bronchial carcinoma (WHO types I–IV) available for detailed study.

### *Treatment*

A total of 5,530/9,090 (61%) patients were given definitive treatment for lung cancer (surgery, radiotherapy, chemotherapy or combinations of these). The proportion of patients given each modality is shown in Table II. Ninety per cent of treated patients received single modality treatment: surgery 40%, radiotherapy 32% and chemotherapy 18%.

Table III shows that the majority (64% overall) of cases of lung cancer received no treatment, taking as the denominator the number of registered cases (with or without a histological diagnosis) each year (total 24,636). The data suggest a trend towards an increased use of radiotherapy from 1981 onwards. The proportion of patients treated by surgery appeared to show a slight fall over the study period. The use of chemotherapy altered very little in terms of the proportion of patients treated although as is seen in a more detailed analysis of SCLC (see below), there were major qualitative changes in the use of this modality. It is important to note that these figures relate to the total number of cases registered and not only to those with histological confirmation of WHO type I–IV tumours.

**Figure 1** Total number of registered lung neoplasms per year for the Mersey Region. Males ■, females ▨, Total —.

**Table I** Proportion of patients treated in each histological category, 1974–86

| *WHO type* | *All* | *Treated* | *Untreated* | *Proportion untreated* |
|---|---|---|---|---|
| I | 4167 (43) | 2682 | 1485 | 36% |
| II | 1909 (19) | 1143 | 766 | 40% |
| III | 1254 (13) | 758 | 496 | 40% |
| IV | 1760 (18) | 947 | 813 | 46% |
| Subtotal | 9090 | 5530 | 3560 | 40% |
| All other | 681 (7) | 386 | 295 | 43% |
| Total | 9771 (100) | 5916 | 3855 | 40% |

Column percentages in parentheses. I, squamous carcinoma; II, small cell carcinoma; III, adenocarcinoma; IV, undifferentiated carcinoma.

**Table II** Treatment used in each histological category, 1974–86

| *WHO type* | *S* | *C* | *R* | *S ± C ± R* | *C + R* | *Total* |
|---|---|---|---|---|---|---|
| I | 1271 (47) | 193 (7) | 997 (37) | 118 (5) | 103 (4) | 2682 (100) |
| II | 143 (13) | 542 (47) | 249 (22) | 82 (7) | 127 (11) | 1143 (100) |
| III | 499 (66) | 61 (8) | 141 (19) | 44 (6) | 13 (1) | 758 (100) |
| IV | 281 (30) | 179 (19) | 361 (38) | 51 (5) | 75 (8) | 947 (100) |
| Totals | 2194 (40) | 975 (18) | 1748 (32) | 295 (5) | 318 (5) | 5530 (100) |

S, surgery; C, chemotherapy; R, radiotherapy. I, squamous carcinoma; II, small cell carcinoma; III, adenocarcinoma; IV, undifferentiated carcinoma. Row percentage in parentheses.

**Table III** Number of patients treated per year expressed as proportion (%) of total number of registered lung neoplasms

| | *S ± C ± R* | *C ± R* | *R* | *Nil* |
|---|---|---|---|---|
| 1974 | 17 | 13 | 10 | 60 |
| 1975 | 14 | 12 | 9 | 65 |
| 1976 | 13 | 13 | 11 | 63 |
| 1977 | 12 | 11 | 13 | 64 |
| 1978 | 11 | 10 | 11 | 68 |
| 1979 | 10 | 11 | 10 | 69 |
| 1980 | 11 | 11 | 10 | 68 |
| 1981 | 9 | 11 | 14 | 66 |
| 1982 | 9 | 9 | 15 | 67 |
| 1983 | 11 | 10 | 15 | 64 |
| 1984 | 10 | 9 | 18 | 63 |
| 1985 | 10 | 10 | 20 | 60 |
| 1986 | 12 | 10 | 17 | 61 |
| Mean | 12 | 11 | 13 | 64 |

S, surgery; C, chemotherapy; R, radiotherapy.

### *Survival by age and sex*

Survival curves for five age bands are shown in Figure 2, which includes all cases, irrespective of whether treatment

**Figure 2** Survival of patients by age at diagnosis: histologically confirmed cases. Number of patients in each group: age 20–39 (□), $n = 44$; 40–49 (+), $n = 326$; 50–59 (◇), $n = 1{,}570$; 60–69 (△), $n = 3{,}280$; ⩾70 (×), $n = 3{,}870$.

was given. The data between 3 and 5 year survival time points have been compressed to improve the resolution of the curves in the region of the median survival time. These curves show a clear relationship between age at diagnosis and survival for lung cancer patients.

There was no significant difference in survival after treatment between males ($n = 4,106$) and females ($n = 1,424$) ($P = 0.4$).

*Survival by histology*

Figure 3 shows the survival curves for treated cases according to histological type. Adenocarcinoma showed the longest survival with a median of 12 months and a 5 year survival of 22%. SCLC patients had a median survival of 6 months following treatment with a 2 year survival of 7.5% and a 5 year survival of 3.5% ($P < 0.001$).

Figure 4 shows similar information for untreated cases and shows the median survival of untreated squamous carcinoma to be 4 months compared to 2 months for SCLC. Adenocarcinoma and undifferentiated carcinoma showed intermediate values. The differences were significant at $P < 0.001$. Less than 1% of SCLC and undifferentiated carcinoma survived 5 years without treatment and less than 5% of squamous carcinoma and adenocarcinoma without treatment. However, 17% of patients with untreated squamous carcinoma were

**Figure 3** Survival of all treated cases according to histological type. Numbers of patients shown in parentheses.

**Figure 4** Survival of untreated cases according to histological type. Number of patients in each group: squamous carcinoma (□), $n = 1,485$; small cell carcinoma (◇), $n = 766$; adenocarcinoma (+), $n = 496$; undifferentiated carcinoma (Δ), $n = 813$.

alive at 1 year from diagnosis. Reasons for treatment not being given were coded by Registry staff and included advanced disease in 56% of those untreated, poor general condition in 19%, a diagnosis at post-mortem in 10%, treatment refusal in 2.5% and others 12.5%. There was no change in survival of untreated patients in any histological category between 1974 and 1986.

*Surgery*

In addition to the 2,194 out of 5,530 cases (40%) undergoing surgery alone, a further 156 (3%) received chemotherapy and 128 (2%) received radiotherapy as well as surgical treatment. Eleven patients (<1%) were treated by all three modalities, giving a total of 2,489 (45%) patients treated by surgery with or without other modalities, the survival of whom is shown in Figure 5. Survival after surgery was similar for squamous carcinoma and adenocarcinoma with median survival 30 and 27 months; 5 year survival 40% and 32.5%, and 10 year survival 25% and 22.5% respectively ($P > 0.05$). The median survival of undifferentiated carcinoma after surgery was 11 months but the 5 year survival was 22.5%. SCLC patients ($n = 225$) showed the shortest overall survival after surgery (10% at 5 years), but in the 40% of these patients who underwent lobectomy 5 year survival was 22.5%.

The survival of these patients was calculated for each of the three periods 1974–77, 1978–81 and 1982–86, showing a continuous improvement in the results after surgery for lung cancer in the Mersey Region between 1974 and 1986. Median survival improved from 13 to 30 months and 5 year survival from 25 to 37.5% (Figure 6, $P < 0.001$). The number of patients treated by surgery in these three periods was 893, 677 and 919 respectively.

Detailed surgical staging was not recorded in the registry data base but Figure 7 shows survival after lobectomy ($n = 1,184$) or pneumonectomy ($n = 1,029$) for all surgical cases in which resection was achieved, and for which the type of operation had been recorded in the Registry. These figures therefore differ from those shown in Table II in relation to surgery and do not include patients undergoing exploratory thoracotomy alone. Patients undergoing lobectomy showed significantly longer survival than pneumonectomy patients:

**Figure 5** Survival of all cases treated by surgery according to histological type. Number of patients shown in parentheses.

**Figure 6** Survival of surgically treated cases (all histological types) for the periods shown. Number of patients shown in parentheses.

**Figure 7** Survival of surgically treated cases (all histological types) 1974–86: lobectomy versus pneumonectomy. Number of patients shown in parentheses.

median 33 versus 16 months, 2 year survival 57.5% versus 42.5% and 5 year survival 37.5% versus 27.5% ($P < 0.001$). These differences were, however, less marked at 10 years.

*Chemotherapy*

A total of 1,460/5,530 (26%) patients received chemotherapy either alone or in combination with another treatment modality, of whom 741 (51%) had a histological diagnosis of SCLC. Median survival following chemotherapy was between 4 and 6 months, with SCLC patients having the longest median survival. The 2 year survival of patients with SCLC treated by chemotherapy was 5% and less than 2.5% survived 5 years. There was a highly significant ($P < 0.001$) improvement in survival after chemotherapy for SCLC during 1974–86 and the 2 year survival rose from 2.5% to 7.5% over this 13 year period. Figure 8 shows the survival curve for SCLC treated by chemotherapy for the period 1982–86 when 408 cases were treated. These changes in survival after chemotherapy between 1974 and 1986 for SCLC were associated with an increase in median survival from 6.5 to 8 months for all treated SCLC patients. The type of chemotherapy used for SCLC changed from single agent cyclophosphamide (93% of chemotherapy used until 1979) to combination chemotherapy (82% of chemotherapy used after 1984) with other single agents constituting less than 10% of all treatments for the entire period of study. The intravenous route was employed in 71% of patients treated with combination regimes and in 27% of those receiving single agents.

The survival of SCLC patients treated by chemotherapy using either single agent or combination chemotherapy is shown in Figure 9. Survival in patients receiving combination chemotherapy was superior to that of patients receiving single agents: median 9 versus 6 months; 2 year survival 10% versus 2.5% ($P < 0.001$).

*Radiotherapy*

The proportion of patients treated by radiotherapy increased during the period of study and radiotherapy was used in 40% of those with histological confirmation representing 13% of all patients with a Registry diagnosis of lung cancer (Table III). The majority of patients treated by radiotherapy were not treated by any other modality (Table II).

Overall survival following radiotherapy showed a median survival of 6 months for SCLC, adenocarcinoma and undifferentiated carcinoma and 8 months for squamous carcinoma. Between 2.5% and 5% of patients survived 5 years following radiotherapy. There was no significant difference in survival after radiotherapy when comparison was made between the periods 1974–77, 1978–81 and 1982–86 ($P > 0.05$). Patients with squamous carcinoma formed the largest number treated by radiotherapy (1,172) and had a median survival of 8 months for each time period. The number of these patients in each period was 247, 303 and 622 respectively.

*Overall survival*

Summary survival curves for all treated cases for 1974–77, 1978–81 and 1982–86 showed a significant although small improvement with time: median survival rose from 8 to 10 months, 2 year survival from 20 to 25% and 5 year survival from 12.5 to 17.5% ($P < 0.001$). These curves therefore give an estimate of the overall impact of the various determinants of outcome, including treatment, on survival in histologically proven bronchial carcinoma in a large Health Region of the United Kingdom. Figure 10 shows survival of all treated cases, irrespective of histological type for the most recent time period (1982–86) and Figure 11 summarises the data for all cases of lung neoplasms in the Mersey Region for the period 1974–86.

**Figure 8** Survival of small cell carcinoma patients treated by chemotherapy, 1982–86 ($n = 408$).

**Figure 9** Survival of small cell carcinoma patients treated by chemotherapy, 1974–86; combination chemotherapy ($n = 183$) versus single agent ($n = 558$).

**Figure 10** Survival of all treated cases of histologically confirmed bronchial carcinoma, 1982–86 ($n$ = 2,518).

### Discussion

Only a small proportion of patients are entered into clinical trials and in a recent survey of 215 new cases of lung cancer referred to McGill University, Canada, significant differences in survival were found between patients treated 'on' or 'off' protocol (Quoix *et al.*, 1986). This paper presents the results of a large population based study in a Health Region of the UK, and has demonstrated the impact of changes in treatment policies, which are not brought out in reports of single or multicentre trials, on account of their selection criteria, relatively small numbers and referral bias (Davis *et al.*, 1985; Carter, 1985).

The quality of data provided by the Merseyside Registry was very high, with very few omissions or errors in each individual patient record. A small number of transposition errors were identified and corrected using the computer software and manual checking of the original Registry data file. Survival time was specifically examined to exclude erroneously long survival times.

The England and Wales Cancer Registration Service is thought to hold the largest registry data set on cancers in the world and obtains its information from Regional Registries such as the MRCR. Completeness of data is probably over 95% and the potential effects of bias and inaccuracy have been estimated to be less than a few per cent (Swerdlow, 1986). These factors, coupled with the effective pathological review at the MRCR, support our contention that the data presented in this study are accurate and consistent, and cover virtually all cases of the disease which actually occurred.

The histological confirmation rate rose overall by 9% between 1974 and 1986, which we feel is attributable to the more widespread availability of fibreoptic bronchoscopy. The number of patients in the United Kingdom undergoing fibreoptic bronchoscopy increased over the same period from 15,000 to 40,000 per annum (Simpson *et al.*, 1986).

The overall distribution of cell types showed the predominance of squamous carcinoma and was in keeping with other published series. The distribution of cell types in the original paper of the Task Force on Lung Cancer was squamous 46%, SCLC 17%, adenocarcinoma 24% and undifferentiated carcinoma 12% (Mountain *et al.*, 1974).

The increase in number of cases of lung cancer in women shown in this study has also been confirmed by others (Andrews *et al.*, 1985). This has been attributed to historical differences in smoking habits between the sexes with women adopting heavy tobacco consumption 20 years later than men. The fall in absolute number of cases in men did not offset the increase in number in women, and the overall number of cases of lung cancer occurring each year in the Mersey Region between 1974 and 1986 did not change. While incidence figures are not presented it is our impression that the observed changes are real rather than due to population shifts, and the likely continued rise in lung cancer incidence in women (Williams, 1987) has important implications for the allocation of health care resources in those areas with a high lung cancer incidence.

The data presented on untreated lung cancer represent the largest available series analysed by histological type. As virtually all untreated cases were shown to have died, the long-term survival rates for treated groups do not require adjustment for errors attributable to incorrect survival time or failure to record death. The changes shown in survival in the treated groups are likely to be a real effect of that treatment, since the untreated patients in each histological category showed numerically no change in survival over the period of study. Assessment of the effect of treatment on squamous cell carcinoma should take account of the 17% 1

**Figure 11** Summary flow chart for all lung neoplasms, Mersey Region, 1974–86.

year survival without treatment. As expected, advanced disease and poor performance status were the major reasons for treatment being withheld (75% of those untreated). We cannot comment on patients who were given active treatment in the absence of a histological diagnosis.

*Surgery*

The TNM system for staging of lung cancer (Mountain, 1986) can only be employed accurately after thoracotomy. In this retrospective survey it is not possible to obtain reliable staging information, or to determine the reason for a treatment decision, including selection criteria for modalities used. Selection for surgery would, however, appear to be appropriate and may have improved over the period of study by greater use of flexible bronchoscopy, isotope scanning and computerised tomography, as well as mediastinoscopy. The 5 year survival rate reported by five British surgeons in a collective series of 8,781 patients was between 25.5 and 26.8% following surgery for bronchial carcinoma and operative mortality was 6% for lobectomy and 12% for pneumonectomy (Belcher, 1983), although the mortality from the latter procedure has improved considerably during the period of this study. The proportion of patients treated by lobectomy has remained at about 50% since the operation was first popularised (Belcher, 1959). Data have not been presented for operative mortality in this study but post-operative deaths are included in the survival curves as cancer deaths.

Surgery for lung cancer has become increasingly safe in recent years as a result of improved preoperative preparation and advances in anaesthesia and intensive care facilities for patients with lung cancer who frequently have co-existing cardiorespiratory diseases (Johnston, 1988). Improvements in surgical techniques, such as the introduction of stapling devices (Kaplan *et al*., 1986) and bronchoplastic procedures, are also possible reasons for the improvement shown in survival after surgery during the period of this study.

*Histology*

Overall, the data confirm previous studies which have shown the relatively good prognosis of adenocarcinoma and squamous carcinoma in terms of 5 year survival. However, survival curves for all histological types show a rapid initial fall within the first 12 months. It is clear that conventional histology does not predict well for this early part of the natural history: it remains to be shown whether DNA ploidy measurement, oncoprotein or growth factor parameters may improve this situation (Carney & De Leij, 1988) and identify subgroups with a higher response to chemotherapy or radiotherapy. Also, to date tumour related prognostic factors are better established for SCLC (Osterlind & Andersen, 1986).

*Treatment*

The results of this study have demonstrated an improvement over time in the survival of patients undergoing surgery for lung cancer. The improved prognosis after chemotherapy for SCLC would appear to justify the increasing intensity, and hence also morbidity, of the treatments employed over the period of study. No similar effect was seen in lung cancer cases treated by radiotherapy, a group comprising predominantly squamous tumours.

Demonstration of an overall improvement in survival in population based series of common tumours has proved elusive over the past 20–30 years, although this has been possible for rarer tumour types including testicular cancer (Boyle *et al*., 1987). There are marked Regional differences in cancer survival data from many causes, with the poorest figures often associated with other parameters of health, such as infant mortality, which are also high in the North of England (Silman & Evans, 1981).

Completeness of registration, stage at presentation, and availability of specialist expertise and treatment facilities may all contribute to these findings. The overall improvements in survival shown here, while modest, strongly suggest an association with more effective treatment for this group of diseases.

We are grateful to Mrs Sandra Gravestock of the MRCR for advice and assistance during this work, and to Dr Kell Osterlind of the Finsen Institute, Copenhagen for help with the survival analyses.

S.W.W. was supported by the Cancer Research Campaign and the Clatterbridge Cancer Research Trust. Some of this work was carried out during a travelling fellowship to the Finsen Institute, Copenhagen.

**References**

ANDREWS, J.L., BLOOM, S., BALOGH, K. & BEAMIS, J.F. (1985). Lung cancer in women: Lahey clinic experience, 1957–1980. *Cancer*, **55**, 2894.

BELCHER, J.R. (1959). Lobectomy for bronchial carcinoma. *Lancet*, **ii**, 639.

BELCHER, J.R.. (1983). Thirty years of surgery for carcinoma of the bronchus. *Thorax*, **38**, 428.

BMDP (1981). Statistical software. University of California Press: Berkeley, CA.

BOYLE, P., KAYE, S.B. & ROBERTSON, A.G. (1987). Changes in testicular cancer in Scotland. *Eur. J. Cancer Clin. Oncol*., **23**, 827.

CAPEWELL, S. (1987). Patients presenting with lung cancer in South East Scotland. *Thorax*, **42**, 853.

CARNEY, D.N. & DE LEIJ, L. (1988). Lung cancer biology. *Semin. Oncol*., **15**, 199.

CARTER, S.K. (1985). Population-based study of small-cell lung cancer (letter). *J. Clin. Oncol*., **3**, 883.

DAVIS, S., WRIGHT, P.W., SCHULMAN, S.F., SCHOLES, D., THORNING, D. & HAMMAR, S. (1985). Long-term survival in small-cell carcinoma of the lung: a population experience. *J. Clin. Oncol*., **3**, 80.

JOHNSTON, M.R. (1988). Selecting patients with lung cancer for surgical therapy. *Semin. Oncol*., **15**, 246.

KAPLAN, D.K., WHYTE, R.I. & DONNELLY, R.J. (1987). Pulmonary resection using automatic stapling devices. *Eur. J. Cardiothor. Surg*., **1**, 152.

MOUNTAIN, C.F. (1986). A new international staging system for lung cancer. *Chest*, **89** (suppl.), 225.

MOUNTAIN, C.F., CARR, D.T. & ANDERSON, W.A.D. (1974). A system for the clinical staging of lung cancer. *Am. J. Roentgenol*., **120**, 130.

OFFICE OF POPULATION CENSUSES AND SURVEYS (1985). *Mortality Statistics: Area*, Series DH5 No. 12. HMSO: London.

OSTERLIND, K. & ANDERSEN, P.K. (1986). Prognostic factors in small cell lung cancer: multivariate model based on 788 patients treated with chemotherapy with or without irradiation. *Cancer Res*., **46**, 4189.

PETO, R., RIKE, M.C., ARMITAGE, R. & 7 others (1977). Design and analysis of randomized clinical trials requiring prolonged observation of each patient. II. Analysis and examples. *Br. J. Cancer*, **35**, 1.

QUOIX, E., FINKELSTEIN, H., WOLKOVE, N. & KREISMAN, H. (1986). Treatment of small-cell lung cancer on protocol: potential bias of results. *J. Clin. Oncol*., **4**, 1314.

SILMAN, A.J. & EVANS, S.J.W. (1981). Regional differences in survival from cancer. *Comm. Med*., **3**, 291.

SIMPSON, F.G., ARNOLD, A.G., PURVIS, A., BELFIELD, P.W., MUERS, M.F., COOKE, N.J. (1986). Postal survey of bronchoscopic practice by physicians in the United Kingdom. *Thorax*, **41**, 311.

SWERDLOW, A.J. (1986). Cancer registration in England and Wales: some aspects relevant to interpretation of the data. *J.R. Stat. Soc*., **149**, 146.

WATKIN, S.W. (1989). Temporal, demographic and epidemiologic variation in histologic types of lung cancer: a literature review. *Lung Cancer*, **5**, 69.
WILLIAMS, C.J. (1987). Preventing lung cancer. *Br. Med. J.*, **295**, 1433.
WORLD HEALTH ORGANIZATION (1978). *International Classification of Diseases*, 8th Revision. WHO: Geneva.
WORLD HEALTH ORGANIZATION (1981). *Histological Typing of Lung Tumours*, 2nd edn. WHO: Geneva.